# 2,3,5,4′-tetrahydroxystilbene-2-O-β-D-glucoside induces autophagy of liver by activating PI3K/Akt and Erk pathway in prediabetic rats

**DOI:** 10.1186/s12906-020-02949-w

**Published:** 2020-06-08

**Authors:** Xuanbin Wang, Jing Zeng, Xiao Wang, Ju Li, Jin Chen, Ning Wang, Miao Zhang, Yibin Feng, Huailan Guo

**Affiliations:** 1 Laboratory of Chinese Herbal Pharmacology, Oncology Center, Renmin Hospital; Hubei Key Laboratory of Wudang Local Chinese Medicine Research; Biomedical Research Institute, Hubei University of Medicine, 39 Middle Chaoyang Road, Shiyan, 442000 Hubei Province China; 2 Department of Traditional Chinese Medicine, Taihe Hospital, Hubei University of Medicine, 32 South Renmin Road, Shiyan, 442000 Hubei Province China; 3 School of Public Health and Management, Hubei University of Medicine, 30 South Renmin Road, Shiyan, 442000 Hubei Province China; 4 School of Chinese Medicine, The University of Hong Kong, 10 Sassoon Road, Pokfulam, Hong Kong, 442000 Hong Kong S.A.R China; 5 Center for Environment and Health in Water Source Area of South-to-North Water Diversion, Hubei University of Medicine, 32 South Renmin Road, Shiyan, 442000 Hubei Province China

**Keywords:** 2,3,5,4′-tetrahydroxystilbene-2-O-β-D-glucoside, Liver injury, Prediabetes, Autophagy

## Abstract

**Background:**

*2,3,5,4′-tetrahydroxystilbene-2-O-β-D-glucoside (TSG) is an active compound derived from Polygonum multiflorum* Thunb., a Chinese Taoist herbal medicine, which exerts lipid lowering, anti-cancer, anti-aging, anti-inflammatory and hepatoprotective effects. However, its role in protecting hepatocytes under pre-diabetic condition remains unclear.

**Methods:**

In this study, we developed prediabetic SD rats by feeding high-fat and high-sugar diet. The body weight, blood lipid, blood glucose, and fasting insulin (FINS) and insulin resistance index (HOMA-IR) were detected and calculated to assess the potential risk of prediabetes. HE and Oil Red O staining was used, and blood level of biochemical index was detected to observe the liver injury. The autophagic cell death-associated signaling proteins, and the potential signaling factors p-Akt/Akt and p-Erk/Erk were detected using western blot to explore the potential effects of TSG on pre-diabetic liver and the underlying mechanisms.

**Results:**

The results showed that the body weight in TSG-treated group was significantly decreased vs. the model group. The blood glucose, the level of FINS and HOMA-IR, TC and TG were decreased in TSG-treated group as well. Furthermore, TSG treatment significantly ameliorated lipid droplet accumulation, enhanced liver anti-oxidative response which may be associated with an increased activity of SOD and GSH-Px, and a decrease of LDLC and MDA. The autophagic cell death-associated proteins, p-AMPK, ATG12, LC3 II, and Beclin 1 were up-regulated in the TSG-treated group, while the upstream signaling pathway, PI3K/Akt and Erk, were activated.

**Conclusions:**

TSG induced liver autophagic cell death to protect liver from prediabetic injury by activating PI3K/Akt and Erk.

**Graphical abstract:**

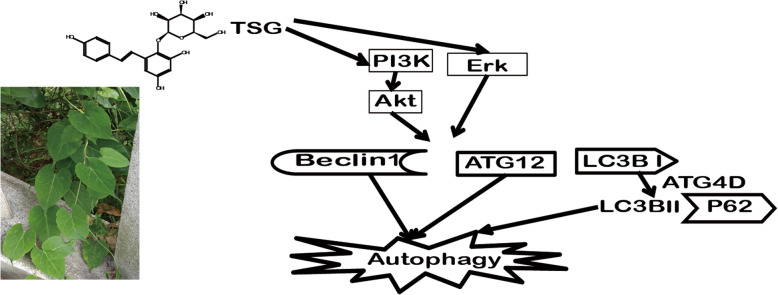

## Highlights

- 2,3,5,4′-tetrahydroxystilbene-2-O-β-D-glucoside is an active compound in Heshouwu
- It may induce autophagy in liver of prediabetic rats
- The underlying mechanisms may be activation of PI3K/Akt and Erk signaling pathways

## Background

Diabetes mellitus (DM) is a metabolic disorder with high blood glucose level and the patients suffer from symptoms of frequent urination, severe thirst and increased appetite. Among all types of DM, type 2 DM (T2DM) is associated with unhealthy lifestyles such as lack of physical exercise, consumption of high-fat and high-sugar food [1]. Terrible chronic complications include visual impairment, cardiovascular disease, stroke, diabetic ketoacidosis, hyperosmolar hyperglycemic state, or even death. The initiation of T2DM may encounter the process of prediabetes. World Health Organization (WHO) defined the condition of intermediate hyperglycemia, which did not go on to develop diabetes, as so-called prediabetes [2]. It was estimated that the overall prevalence of prediabetes was up to 35.7% in China in 2013 [3]. The prediabetes was defined as impaired fasting glucose (IFG) and impaired glucose tolerance (IGT) and/or HbA_1c_ risk [4]. Although symptoms of prediabetes may not be obvious, IFG and IGT might result in reversible/gentle liver injury, and in consequence lead to diabetes [5]. Furthermore, prediabetes may increase the risk of kidney diseases, cardiovascular diseases, liver disorders and all-cause mortality.

Progress of prediabetic condition in liver may encounter a risk in lipoperoxidation, nonalcoholic fatty liver disease, or even liver cancer [6]. Among these, lipoperoxidation results from excessive accumulation of triglyceride in hepatocytes by endoplasmic reticulum (ER) stress [7]. ER stress can initiate IRE1-XBP1, PERK-eIF2α-ATF4 and ATF6 signaling pathway, up-regulate GRP78, CHOP and GADD34, and consequently evoke apoptosis, or apoptosis and autophagy simultaneously [8]. Some intersection signaling proteins of apoptosis and autophagy, Bcl-2, Beclin 1, and ATG4D, for instance, would be activated [9]. This may be the one of the evidence that autophagy plays a key role in protecting cells in response to stress [10]. Prevention and reversal of prediabetes attracts more and more attentions by clinical and laboratory scientists, including Chinese medicine experts and practitioners.

**Fig. 1:**
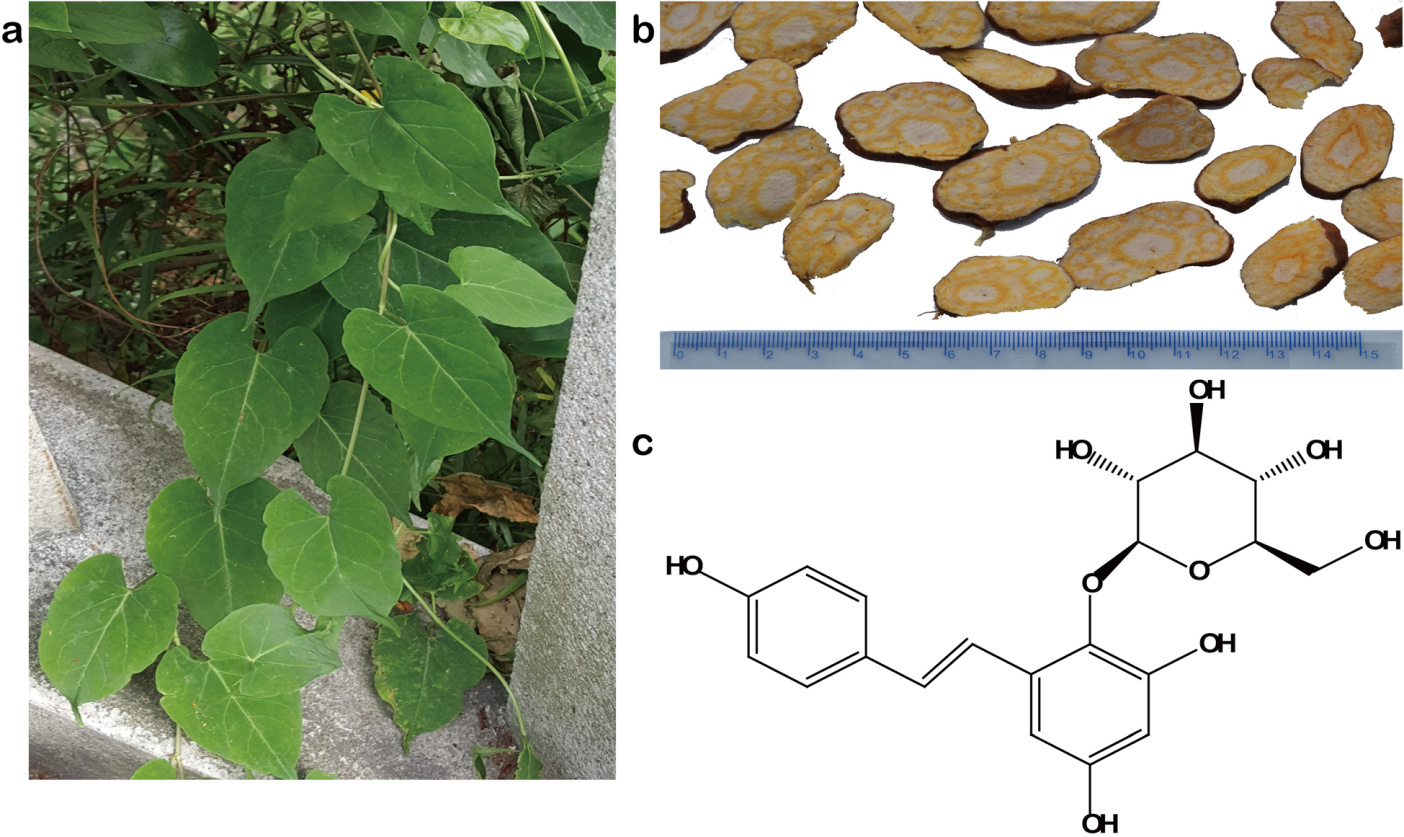
2,3,5,4′-tetrahydroxystilbene-2-O-β-D-glucoside (TSG) is the main active compound in root of Heshouwu (*Polygonum multiflorum* Thunb.). **a** Plant of Heshouwu. **b** Radix slices of Heshouwu from where 2,3,5,4′-tetrahydroxystilbene-2-O-β-D-glucoside (TSG) was derived. **c** Structure of TSG

*2,3,5,4′-tetrahydroxystilbene-2-O-β-D-glucoside* (TSG) is the main active compound in a Chinese medicinal herb, *Heshouwu (Polygonum multiflorum* Thunb.) [11]. Previous studies showed that it exerted anti-hyperlipidemia, anti-atherosclerosis, treating Alzheimer’s disease (AD) and Parkinson’s disease (PD), hepatoprotection and anticancer [11–13] (Fig. 1). TSG protected PC12 cells from apoptosis [11] and promoted murine pre-osteoblastic MC3T3-E1 cell proliferation and differentiation by activating PI3K/Akt [14]; TSG suppressed fibronection, CTGF, TGF-β, and MCP-1 and effectively prevented renal injury in diabetic nephropathy [15]; TSG exerted anti-diabetic gastrointestinal dysmotility by activating Erk and up-regulating PPAR γ, SIRT1 [16] and TGF-β1 *in vivo* [17]; TSG also ameliorated diabetes by reducing human serum advanced glycation end products (AGEs) [18]*.* However, it is still unclear if TSG attenuates liver injury in prediabetes.

In this study, we developed a high-fat and high-sugar fed rat model and observed the effects of TSG against liver injury in prediabetic rats. The aim was to investigate if TSG could improve prediabetic liver, and explore underlying mechanisms.

## Methods

### Reagents and antibodies

TSG was purchased (batch no. 110844–201,109) from the National Institute for the Control of Pharmaceutical and Biological Products (Beijing, China). Metformin (Met) was purchased from Sino-American Shanghai Squibb Pharmaceuticals Ltd. (Shanghai, China). Primary antibodies including microtubule inositol 3 kinase p85 (PI3K, catalog no.4228), phospho-serine-threonine kinase (p-Akt, serine 473, #406), phospho- extracellular regulated protein kinases p44/42 (Erk, Thr202/Tyr204, #9106), SQSTM1/p62 (#88588), adenosine 5′-monophosphate (AMP)-activated protein kinase (AMPK, # 2532), autophagy-related protein 12 (ATG12, #4180), Beclin-1 (#3495), B-cell lymphoma-2 (Bcl-2, #3869), and microtubule-associated light chain 3 (LC3, #L7543) were purchased from Cell Signaling Technology (Danvers, MA, USA). Phospho-inositol-requiring enzyme-1a (p-IRE1a, #PA5–85647) was purchased from Thermo Fisher Scientific (China) Company (Shanghai, China). Antibodies against total serine-threonine kinase (Akt, catalog no. sc-9272) and Erk (catalog no. sc-154), activating transcription factor 6 (ATF-6, catalog no. sc-22,799), glucose-regulated protein 78 (GRP 78, catalog no. sc-13,968), growth arrest and DNA damage-inducible protein (GADD34, catalog no. sc-794) and C/EBP homologous protein (CHOP, catalog no. sc-71,136) were purchased from Santa Cruz Biotechnology, Inc. (Santa Cruz, CA, USA). Autophagy-related protein 4D (ATG4D, catalog no. #NBP2–41308) was purchased from Novus Biologicals (Littleton, CO, USA). Electrochemiluminescence (ECL), Polyvinylidene difluoride (PVDF) membranes were purchased from Millipore Pierce Biotechnology (Rockford, IL, USA). Bicinchoninic acid (BCA) protein assay kits were obtained from Beyotime Institute of Biotechnology (Shanghai, China). Reagent kits used for superoxide dismutase (SOD) activity, glutathione peroxidase (GSH-Px) activity and malondialdehyde (MDA) in serum were purchased from Nan Jing Jian Cheng Biotechnology Co., Ltd. (Nanjing, China). Other materials were all obtained commercially and were of analytical grade.

### Animals and prediabetes model

Thirty eight 8-week-old male SD rats (150 g - 180 g, specific pathogen free) were purchased from Hunan SJA Laboratory Animal Co., Ltd. (Changsha, China). All animal experiments conformed to the British Home Office Regulations (Animal Scientific Procedures Act 1986) for the care and use of animals. The animal protocol was approved by the Animal Care and Use Committee of Hubei University of Medicine (No.2014–1).

To develop the prediabetic model animals, the methodology followed the previous study with minor modifications [19]. Briefly, the rats were housed at 23 ± 2 °C with humidity of 55 ± 10%, under a 12:12 h light-dark cycle with food and water *ad libitum* in the specific pathogen free (SPF) cages before administration*.* After one week of acclimatization, the rats were randomized to divided into control group (Ctrl; *n* = 8) with feeding routine chow diet, and prediabetic group (*n* = 30) with high-fat and high-sugar dieting for 12 weeks. The routine chow diet for the Ctrl group contained an energy of 3.2 kJ/g (fat, carbohydrate and protein accounted for calories 10.2, 23.3 and 66.5%, respectively); The prediabetic group were fed with high-fat diet which contained an energy of 5.2 kJ/g (fat, carbohydrate and protein accounted for calories 56.0, 37.0 and 7%, respectively) including 20% lard, 7% egg yolk powder, 5% sucrose, and 68% basal feed. The prediabetic animal model was successfully established in 24 out of 30 rats. Then the 24 rats were randomly divided into 3 groups, Model, TSG-treated group (TSG) and metformin-treated group (Met, positive control).

**Fig. 2:**
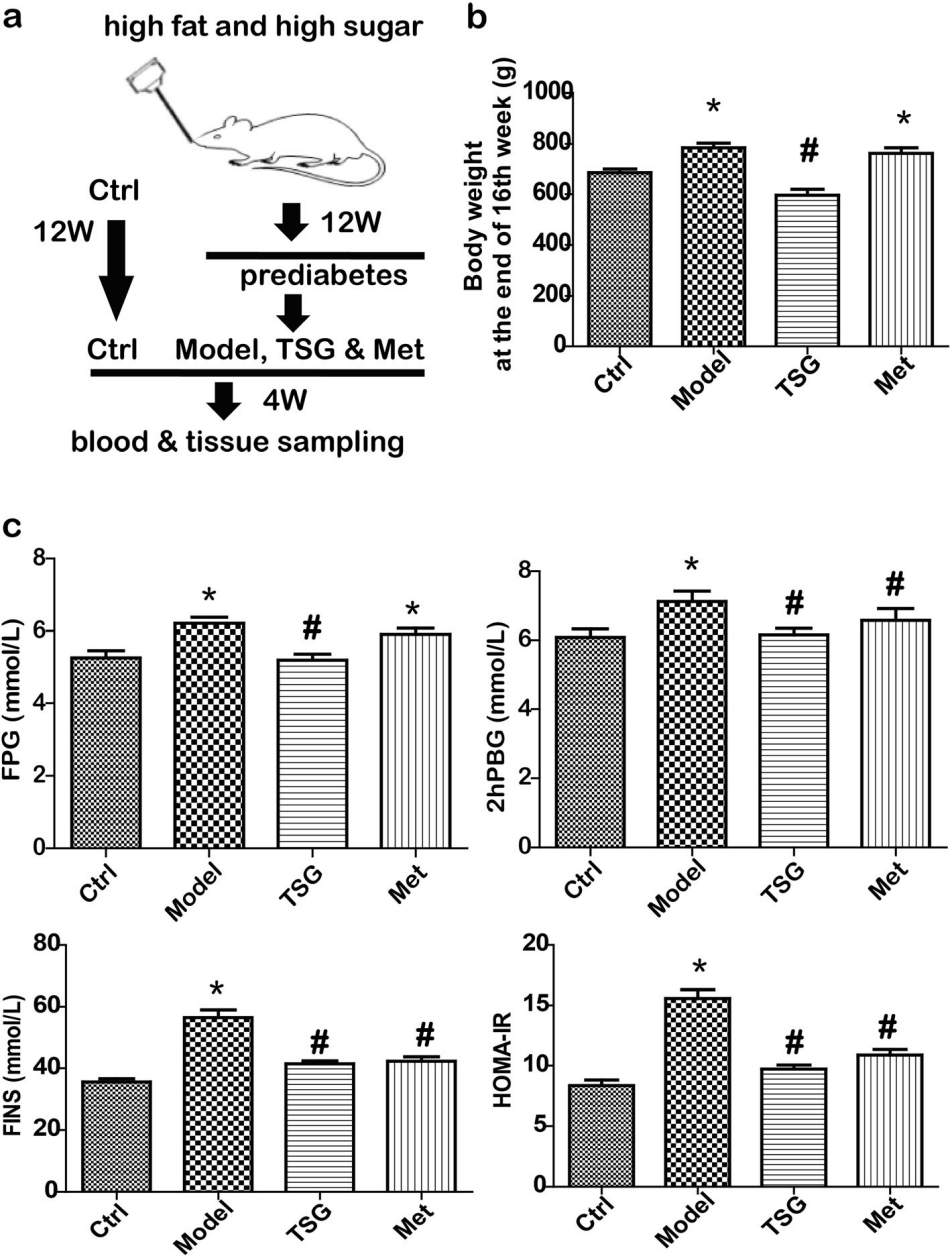
TSG decreased body weight and the level of blood glucose in prediabetic rats. **a** Process of TSG administration in prediabetic rats. After establishment of prediabetic animal model, rats were administrated with 0.9% sodium chloride solution (the control and model groups), TSG (TSG group) and metformin (Met group, positive control), respectively. **b** TSG reduced body weight compared with the model group. **c** TSG decreased the level of FPG, 2hPBG, FINS and HOMA-IR. Ctrl: the control group (*n* = 8); Model: the model group (n = 8); TSG: the TSG-treated group (*n* = 8); Met: the Met-treated group (*n* = 8). *: *P* < 0.05 vs. Ctrl; #: *P* < 0.05 vs. Model

For the drug intervention, TSG and Met groups were given TSG (i.g. with 100 mg/kg/day, once daily) and Met (i.g. with 200 mg/kg/day, once daily), respectively, while both of the Ctrl group and Model group were given 5 mL of 0.9% sodium chloride solution (once daily). The weight of food accounted for 3% of body weight (BW). Then body weight, food intake, and blood glucose from the caudal vein was monitored weekly. At the end of the 12th week, the diabetic index, fasting plasma glucose (FPG) was tested after an 8 h fasting, and two-hour postprandial blood glucose (2hPBG) was conducted using intragastric administration with 50% glucose solution 2 g/kg body weight. When the experiments had lasted for another four weeks (Fig. 2a), FPG and 2hPBG were measured again. The next day, the rats were anesthetized using pentobarbital (40 mg·kg^− 1^, i.p.) [20]. The blood was sampled for metabolic characterization assay. The livers were sampled and stored at − 80 °C until use. Finally, all rats were sacrificed with carbon dioxide (CO_2_) inhalation according to the protocol of the Experimental Animal Center of Hubei University of Medicine. Briefly, each rat was normally placed in a polycarbonate chamber. CO_2_ was emitted into chamber at a flow rate of about 5.5–7.5 L/min until the rat was unconscious. Then the flow of CO_2_ continued for at least 60 s to ensure that the breath was not seen before removing the rat from the chamber. The rat bodies were collected and given a laboratory animal harmless treatment by the Experimental Animal Center of Hubei University of Medicine.

### Metabolic characterization assay

SOD, MDA and GSH-Px, in serum were measured using the Metabolic Assay Kits (Nanjing Jiancheng Company, Nanjing, China). FPG, 2hPBG and fasting insulin (FINS) in blood were detected using Rat/Mouse Insulin Enzyme-Linked Immunosorbent Assay (ELISA) Kit (Merck-Millipore, Shanghai, China). The index of insulin resistance (HOMA-IR) was calculated with a formula of HOMA-IR = FINS×FPG/22.5.

### Liver pathology and lipid analysis

To detect liver condition change, plasma total cholesterol (TC), triglyceride (TG), high-density lipoprotein (HDLC) and low-density lipoprotein (LDLC), were detected using the fully automatic biochemical analyzer (ADVIA1800, Siemens Healthcare Diagnostics Inc., Germany) [5]. Hematoxylin and eosin (HE) staining was conducted in paraffin-embedded sections fixed with paraformaldehyde according to the manufacturer’ s instructions. Furthermore, Oil Red O staining of liver frozen sections was conducted to assess lipid deposition. Briefly, the samples were incubated with 0.5% Oil Red O solution at 60 °C for 8 min, and washed with 85% isopropanol and double distilled water (DD water). Hematoxylin was used for staining for 1.5 min followed by DD water washing again. The red or orange stood for lipid [21].

### Western blot

The liver samples were homogenated and hydrolyzed. The total proteins were collected and BCA was used for detecting the concentration of proteins. The proteins were separated using running buffer and transferred into membranes. After incubated with primary antibodies and second antibodies, the protein ladders were filmed with ECL and the fluorescent bands were visualized using Chemic Dox XRS variable mode imager (Bio-RAD, Hercules, USA). The protein level was calculated using ImageJ 1.38x and analyzed using GraphPad Prism 5.01.

### Statistical analysis

Data were presented as mean ± S.E. and analyzed by one-way analysis of variance (one-ANOVA) with LSD multiple comparison test using SPSS software package (version 22.0) (SPSS, Chicago, IL, USA). *P* < 0.05 was considered statistically significant.

## Results

### TSG reversed the increase of body weight, blood glucose, and the level of FINS and HOMA-IR

As shown in Fig. 2, the body weight, FPG and 2hPBG, FINS and HOMA-IR were increased in Model group vs. Ctrl group, indicating high-fat and -sucrose feeding increased body weight and blood glucose in the prediabetic rats. In contrast, TSG reversed the increased level of body weight, FPG, 2hPBG, FINS and HOMA-IR, indicating TSG attenuated the prediabetic condition in the rats as compared to Model group.

### TSG reduced hepatic lipid biosynthesis and enhanced the capacity of anti-oxidative stress

**Fig. 3:**
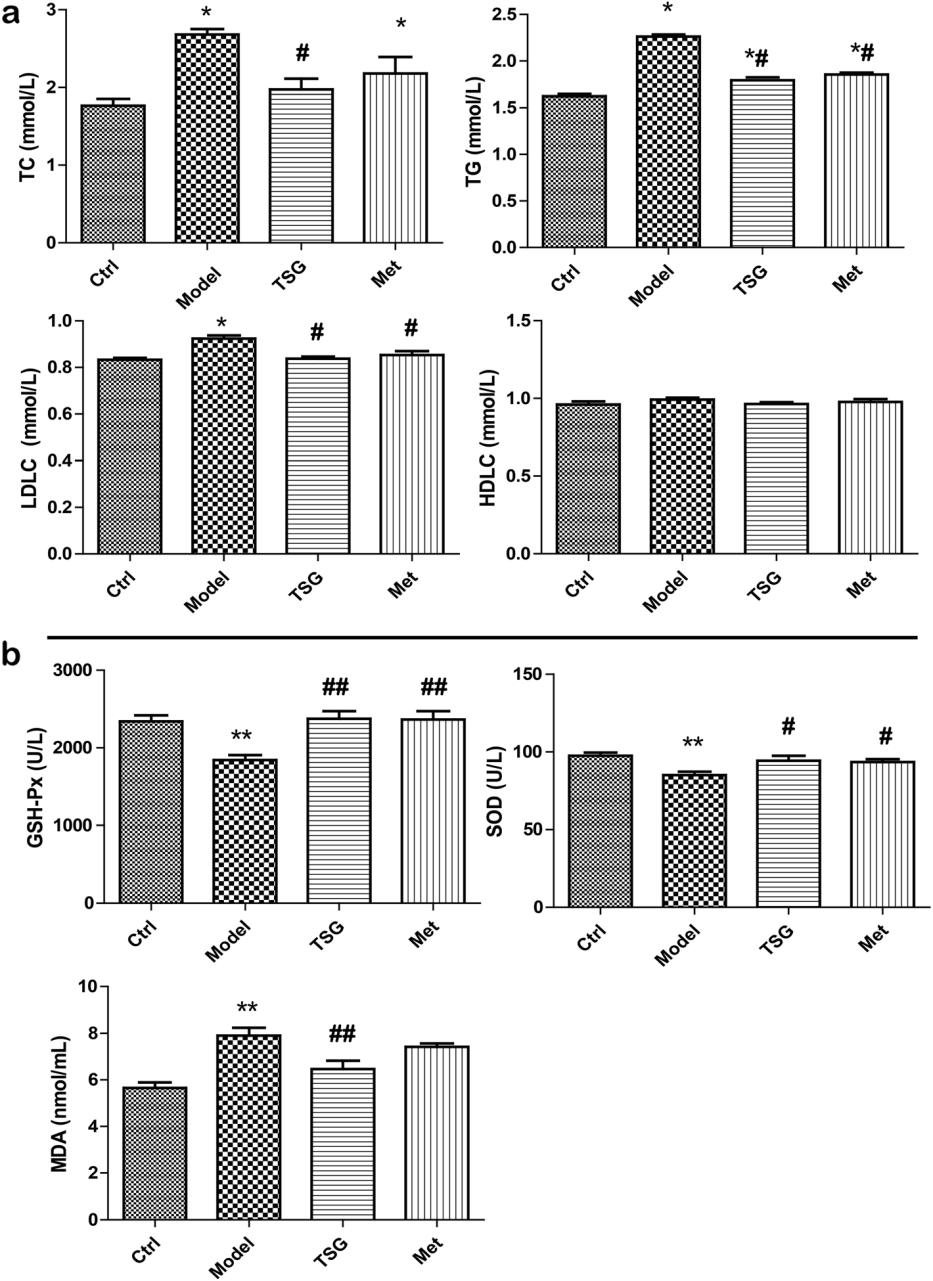
TSG reduced hepatic lipid biosynthesis. **a** Compared with the model group, TSG decreased the blood level of TC, TG and LDLC in rats, while there was no change in HDLC. **b** Compared with the model group, TSG activated GSH-Px and SOD, and decreased the level of MDA in rat blood. Ctrl: the control group (*n* = 8); Model: the model group (*n* = 8); TSG: the TSG-treated group (*n* = 8); Met: the Met-treated group (*n* = 8). *: *P* < 0.05 vs. Ctrl; #: *P* < 0.05 vs. Model; ##: *P* < 0.01 vs. Model

A homeostatic function of liver requires a normal antioxidant defense system [22]. To observe the effects of TSG on lipid accumulation and lipoperoxidation, we detected the level of TC, TG, LDLC and HDLC, and the activation of GSH-Px and SOD and the level of MDA. Compared with Model group, TSG reduced the level of TC, TG, LDLC and MDA, activated the enzymes GSH-Px and SOD in TSG group (Fig. 3).

TSG ameliorated lipid droplet accumulation and slight liver injury without apoptosis in prediabetic rats.

**Fig. 4:**
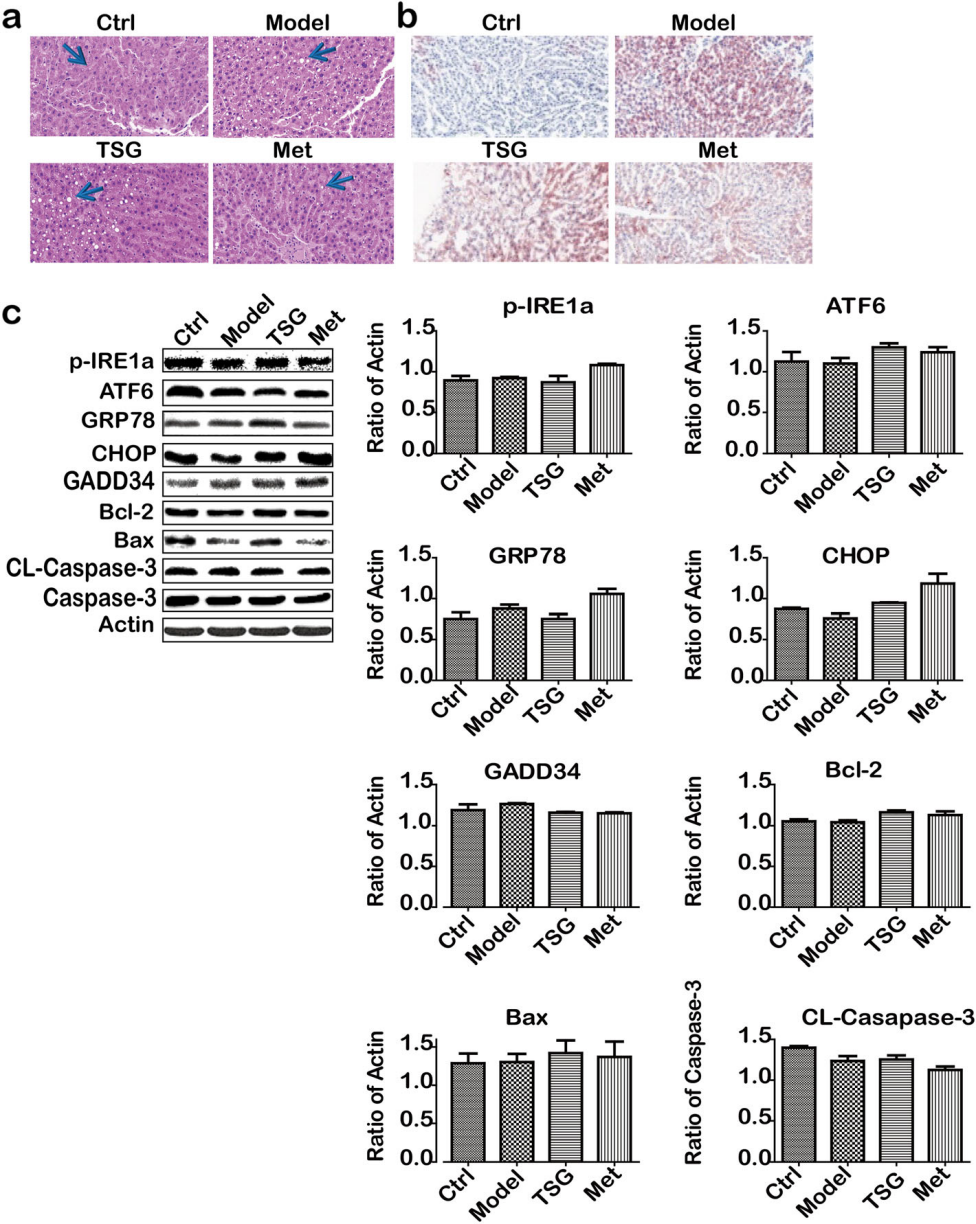
TSG ameliorated liver slight injury without apoptosis in prediabetic rats. **a** HE staining. Arrow-pointed dots (white) stand for accumulated lipid droplets; **b** Oil Red O staining. Red means lipid droplet accumulation. **c** Apoptotic-associated proteins were no changed in all groups. Ctrl: the control group (*n* = 8); Model: the model group (*n* = 8); TSG: the TSG-treated group (*n* = 8); Met: the Met-treated group (*n* = 8)

To evaluate the role of TSG in the liver injury in prediabetic rats, hematoxylin-eosin (HE) staining, Oil Red O staining, and western blotting for the liver tissue were performed and measured. HE staining and Oil Red O staining showed that compared with Model group, TSG decreased the excessive fatty droplet accumulation and the slight degree of liver injury (Fig. 4a).

Since lipid accumulation and excessive lipoperoxidation may lead to the activation of endoplasmic reticulum (ER) stress proteins, IRE1a, ATF6, GRP78, CHOP and GAD34, etc. [23], the cause of up-regulation of apoptosis, we conducted western blotting to observe the potential role of TSG in prevention against apoptosis. Interestingly, the data showed that in neither Model group nor TSG and Met groups there was significant change in ER-stress associated proteins and apoptosis-associated proteins (Fig. 4b), indicating there is no apoptosis in prediabetic stage.

### TSG induced autophagic cell death in the prediabetic liver cells

**Fig. 5:**
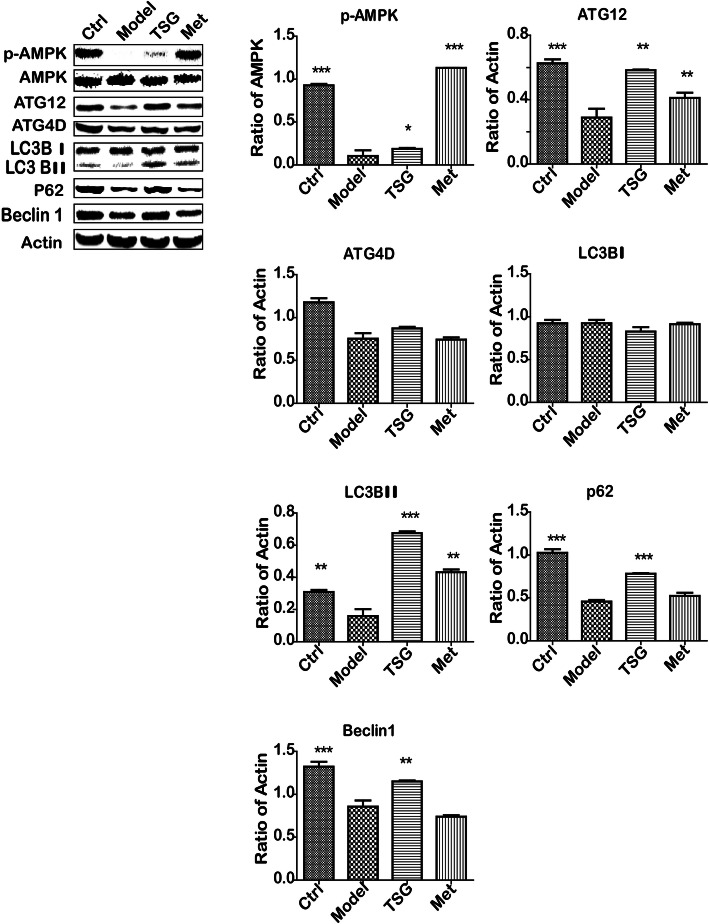
TSG induced liver autophagic cell death. Protein expression in the Ctrl, Model, TSG and Met Group. Ctrl: the control group (*n* = 8); Model: the model group (*n* = 8); TSG: the TSG-treated group (*n* = 8); Met: the Met-treated group (*n* = 8). *: *P* < 0.05 vs. Model; **: *P* < 0.01 vs. Model; ***: *P* < 0.001 vs. Model

Autophagy plays an essential protective role in cells encountering the stress. To investigate if TSG induced autophagy in liver, autophagy-associated proteins on signaling pathways were detected. The results showed that p-AMPK, ATG12, LC3 II and Beclin 1 were up-regulated in the TSG-treated group, indicating that autophagic cell death of the liver cells occurred (Fig. 5).

### TSG induced autophagy in prediabetic rats by activating PI3K/Akt and Erk

**Fig. 6:**
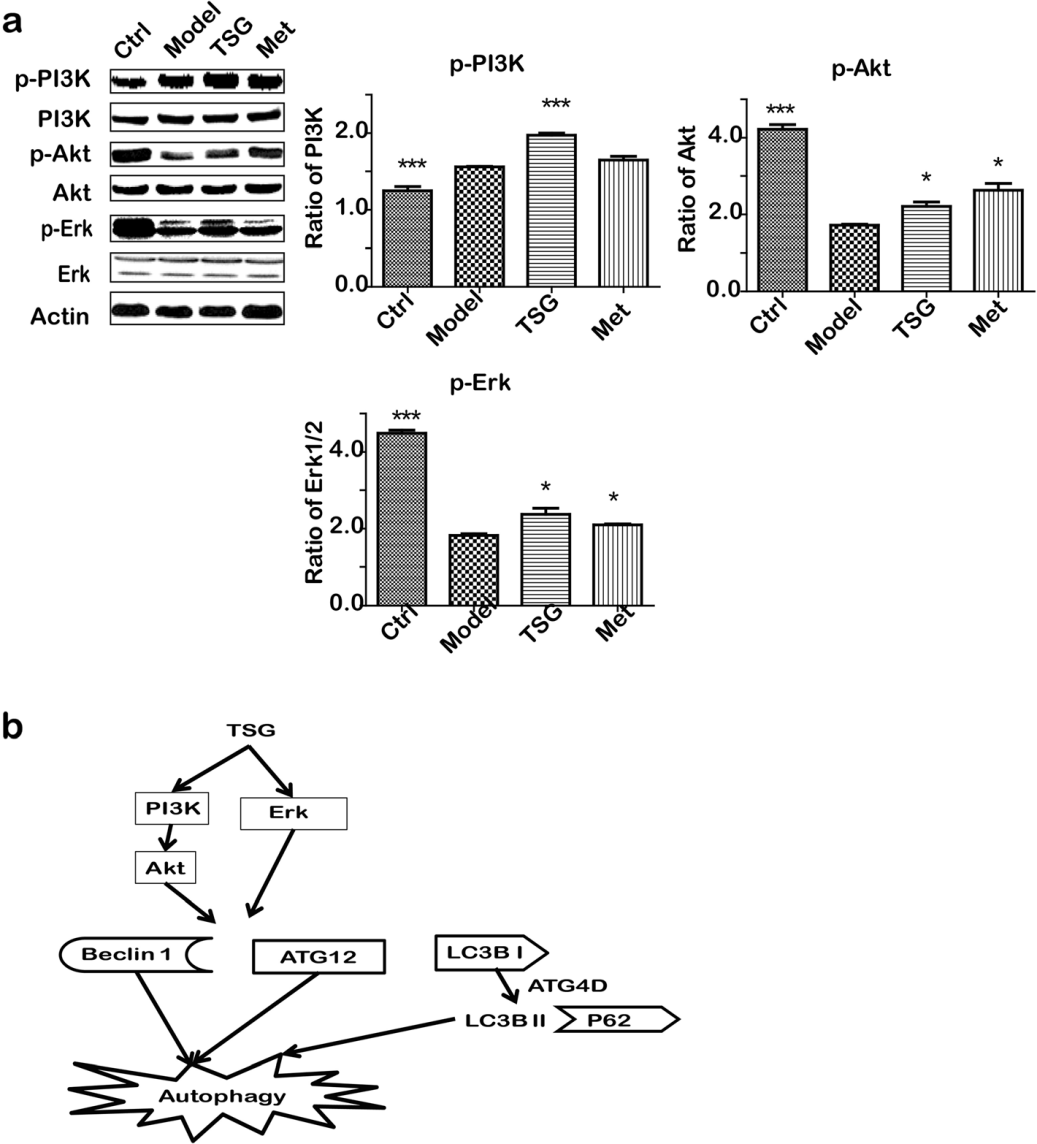
TSG induced liver autophagic cell death by activating PI3K/Akt and Erk. **a** The expression of autophagic cell death-associated proteins. **b** The potential autophagy-associated pathway of TSG-treated hepatocytes. Ctrl: the control group (*n* = 8); Model: the model group (*n* = 8); TSG: the TSG-treated group (*n* = 8); Met: the Met-treated group (*n* = 8). *: *P* < 0.05 vs. Model; ***: *P* < 0.001 vs. Model

PI3K/Akt is a transcriptional pathway that plays a crucial role in diabetes [24]. Furthermore, Erk also induces prediabetic hepatocyte injury and results into ER stress-mediated apoptosis [7] and/or autophagy [25]. Akt and Erk mediates ER stress by binding of GRP78 [26]. To explore the protective mechanisms of TSG on liver, we detected PI3K/Akt, Erk and their phosphorylated protein level in this study. We found that TSG treatment significantly activated PI3K, Akt and Erk by enhancing the phosphorylation of the proteins, indicating TSG might induce autophagy to protect liver from stress by activating PI3K/Akt and Erk signaling pathway (Fig. 6).

## Discussion

Prediabetes, the condition between normal state and T2DM may not have obvious symptoms but is usually accompanied with IFG and IGT, and/or HbA_1c_ disorder. Additionally, regarding the thresholds for diagnosis of prediabetes, there is no global consensus in scientific society. WHO defined prediabetes as IFG with a FPG threshold of 6.1–6.9 mmol/L (1100–1250 mg/L) and IGT with a 2hPBG threshold of 7.8–11.0 mmol/L (1400–1990 mg/L), while American Diabetes Association (ADA) required IFG of 5.6–6.9 mmol/L (1000–1250 mg/L) and IGT of 7.8–11.0 mmol/L (1400–1990 mg/L), and HbA_1c_ of 5.7–6.4% (39–46 mmol/mol). International Expert Committee (IEC) documented HbA_1c_ with a threshold of 6.0–6.4% (42–46 mmol/mol) [4]. Given HbA_1c_-based diagnosis of prediabetes has gender and race specification than that of fasting glucose concentration [4], we used IFG and IGT as the diagnosis index to define the prediabetic condition.

The potential life-threatening risk and upset prognosis make it necessary to prevent prediabetes. Though physical exercise and dieting are good for prevention and treatment of prediabetes, drug intervention is the alternative way for people unwilling and/or not suitable to exercise and diet.

High blood glucose during prediabetic condition in liver can lead to lipoperoxidation, nonalcoholic fatty liver disease, or even liver cancer [6]. Among these, lipoperoxidation results from excessive accumulation of triglyceride in hepatocytes by endoplasmic reticulum (ER) stress [7]. ER stress can initiate IRE1-XBP1, PERK-eIF2α-ATF4 and ATF6 signaling pathway, up-regulate GRP78, CHOP and GADD34, and consequently evoke apoptosis. However, at the stage of prediabetes, there may be no apoptosis or, only apoptosis and autophagy simultaneously occur [8]. Thus some intersection signaling proteins of apoptosis and autophagy, Bcl-2, Beclin 1, and ATG4D, for instance, would be activated [9]. This may be the one of the evidence that autophagy plays a key role in protecting cells in response to stress [10].

Chinese medicines (CMs) have been used to treat diseases for twenty centuries, some of which can ameliorate DM as well as prediabetes [27]. TSG is a natural product derived from a Chinese medicinal herb, *Heshouwu*. Evidence showed that TSG had been used for anti-diabetes in different organs [11], However, it is still unclear if TSG could be used for prediabetic liver injury. In this study, the data showed that TSG reduced body weight, FPG and 2hPBG. Furthermore, level of FINS and HOMA-IR decreased in TSG-treated group, indicating TSG may be used for decreasing the risk for prediabetes. We also found TSG attenuated liver injury in prediabetic rats by activating SOD and GSH-Px and decreasing TG, TC, LDLC and MDA level. To observe hepatocyte injury, the cell death factors including apoptosis and autophagy cascade-associated proteins were detected by western blotting. Although an impaired activation of SOD and GSH-Px and an elevated MDA level, and lipid accumulation in liver, no ER-stress apoptosis occurred in hepatocytes. Because there was no change in the expression of ER-associated apoptotic proteins (p-IRE1a, ATF6, GRP78, CHOP, GADD34, Bcl-2, Bax and Caspase-3), indicating there was no apoptosis in prediabetic liver.

However, interestingly, we found that p-AMPK, ATG12, LC3 II and Beclin 1 were up-regulated in the TSG-treated group, indicating TSG induced autophagic cell death in prediabetic liver (Fig. 6b). This means that there may be autophagic cell death instead of apoptosis in the stage of prediabetes and TSG could decrease such a risk. To explore the underlying mechanisms of effects of TSG on liver lipid accumulation, lipid oxidative stress and autophagy, the potential signaling pathways were detected by western blot. Since PI3K/Akt is a key transcriptional pathway that plays a crucial role in both diabetes and autophagic cell death [24], activation of PI3K and the downstream protein Akt can alleviate diabetic cellular metabolic flux [28]. On the other hand, activation of PI3K and Erk may also result in autophagy by up-regulating Beclin 1 and LC-3 signaling pathway [25]. In our results, TSG treatment may initiate the procedure of PI3K/Akt and Erk-mediated autophagy. This may be the one of the new evidence for the protective role of autophagy from other cell deaths, especially apoptosis. The result might be contradictory to the previous study in which TSG suppressed the mesenteric vascular autophagy in the male Zucker diabetic fatty rats by activating Akt [29]. This difference might result from a different phase of the disorder, different target organs, diverse methodology and/or experiments.

As to the dosage of TSG, the previous studies showed that TSG could improve hypoglycemia [30], enhance digestive [16] and kidney function [15] in diabetic mice with a dosage range from 10 to 100 mg/kg, and be used for anti-diabetic nephropathy in rats with a dosage range from 10 to 20 mg/kg. In this study, we used 100 mg/kg of TSG based on the previous studies and our preliminary test.

Regarding the pharmacokinetic approaches to TSG, it is reported that the oral bioavailability was a range from 0.9 to 22.46%. It is about 6–9 min for TSG to reach the maximum concentration [31], indicating TSG might be a promising potential drug candidate for clinical application.

For the limitation of this study, the relationship between PI3K/Akt and ERK pathway and autophagy should be further clarified. Also, the linkage of ATF6, GPR78, CHOP, the endoplasmic reticulum stress signals and lipid accumulation should be investigated. For TSG, there are still some problems to be explained, e.g. TSG bioavailability after chronic administration to rats should be studied in the future.

## Conclusions

Collectively, TSG might induce autophagy which attenuates mild liver injury by activating PI3K/Akt and Erk signaling pathways, which deserves further studies.

## Data Availability

The datasets supporting the conclusions of this article are included within the article and its additional files.

## Abbreviations

TSG: 2,3,5,4′-tetrahydroxystilbene-2-O-β-D-glucoside
Met: Metformin
FINS: Fasting insulin
HOMA-IR: Insulin resistance index
HE: Staining, hematoxylin-eosin staining
SOD: Superoxide dismutase
GSH-Px: Glutathione peroxidase
TG: Triglyceride
TC: Total cholesterol
LDLC: Low-density lipoprotein
HDLC: High-density lipoprotein
MDA: Malondialdehyde
DM: Diabetes mellitus
T2DM: Type 2 DM
IFG: Impaired fasting glucose
IGT: Impaired glucose tolerance
OGTT: Oral glucose tolerance test
FPG: Fasting plasma glucose
2hPBG: Two-hour postprandial blood glucose
HCC: Hepatocellular carcinoma
AD: Alzheimer’s disease
PD: Parkinson’s disease
ATG12: Autophagy-related protein 12
ATG4D: Autophagy-related protein 4D
LC3 I: Microtubule-associated light chain 3 I
LC3 II: Microtubule-associated light chain 3 II
Bcl-2: B-cell lymphoma-2
ECL: Enhanced chemiluminescence
ELISA: Enzyme-linked immunosorbent assay
ER: Endoplasmic reticulum
IRE1a: Inositol-requiring enzyme-1a
ATF6: Activating transcription factor 6
GRP78: Glucose-regulated protein 78
CHOP: C/EBP homologous protein
GADD34: Growth arrest and DNA damage-inducible protein
i.g.: Intragastric administration
i.p.: Intraperitoneal administration

## References

[1] Zhao Q, Laukkanen JA, Li Q, Li G (2017). Body mass index is associated with type 2 diabetes mellitus in Chinese elderly. Clin Interv Aging.

[2] Edwards CM, Cusi K (2016). Prediabetes: a worldwide epidemic. Endocrinol Metab Clin North Am.

[3] Wang L, Gao P, Zhang M, Huang Z, Zhang D, Deng Q, Li Y, Zhao Z, Qin X, Jin D (2017). Prevalence and ethnic pattern of diabetes and Prediabetes in China in 2013. JAMA.

[4] Makaroff LE (2017). The need for international consensus on prediabetes. Lancet Diabetes Endocrinol.

[5] Burgeiro A, Cerqueira MG, Varela-Rodriguez BM, Nunes S, Neto P, Pereira FC, Reis F, Carvalho E. Glucose and Lipid Dysmetabolism in a Rat Model of Prediabetes Induced by a High-Sucrose Diet. Nutrients. 2017;9(6):638. 10.3390/nu9060638 PMC5490617 28635632

[6] Xu WG, Qian YF, Wu J (2017). The effect of prediabetes on hepatocellular carcinoma risk: a systematic review and meta-analysis. Minerva Med.

[7] Lin MH, Yen JH, Weng CY, Wang L, Ha CL, Wu MJ (2014). Lipid peroxidation end product 4-hydroxy-trans-2-nonenal triggers unfolded protein response and heme oxygenase-1 expression in PC12 cells: roles of ROS and MAPK pathways. Toxicology.

[8] Galluzzi L, Pietrocola F, Levine B, Kroemer G (2014). Metabolic control of autophagy. Cell.

[9] Mukhopadhyay S, Panda PK, Sinha N, Das DN, Bhutia SK (2014). Autophagy and apoptosis: where do they meet?. Apoptosis.

[10] Mauro C, Silvia C (2016). Autophagy inhibition and mitochondrial remodeling join forces to amplify apoptosis in activation-induced cell death. Autophagy.

[11] Lin L, Ni B, Lin H, Zhang M, Li X, Yin X, Qu C, Ni J (2015). Traditional usages, botany, phytochemistry, pharmacology and toxicology of Polygonum multiflorum Thunb.: a review. J Ethnopharmacol.

[12] Li H, Cao S, Wang X, Zuo Q, Chen P, Liu Y, Liu M, Feng Y, Hao X, Xiang L (2016). Quality evaluation of Heshouwu, a Wudang Taoist medicine in China. Exp Ther Med.

[13] Li H, Wang X, Liu Y, Pan D, Wang Y, Yang N, Xiang L, Cai X, Feng Y (2017). Hepatoprotection and hepatotoxicity of Heshouwu, a Chinese medicinal herb: context of the paradoxical effect. Food Chem Toxicol.

[14] Fan YS, Li Q, Hamdan N, Bian YF, Zhuang S, Fan K, Liu ZJ. Tetrahydroxystilbene Glucoside Regulates Proliferation, Differentiation, and OPG/RANKL/M-CSF Expression in MC3T3-E1 Cells via the PI3K/Akt Pathway. Molecules. 2018;23(9):2306. 10.3390/molecules23092306 PMC6225160 30201908

[15] Chen GT, Yang M, Chen BB, Song Y, Zhang W, Zhang Y (2016). 2,3,5,4′-Tetrahydroxystilbene-2-O-beta-d-glucoside exerted protective effects on diabetic nephropathy in mice with hyperglycemia induced by streptozotocin. Food Funct.

[16] Chang MJ, Xiao JH, Wang Y, Yan YL, Yang J, Wang JL (2012). 2, 3, 5, 4′-Tetrahydroxystilbene-2-O-beta-D-glucoside improves gastrointestinal motility disorders in STZ-induced diabetic mice. PLoS One.

[17] Li C, Cai F, Yang Y, Zhao X, Wang C, Li J, Jia Y, Tang J, Liu Q (2010). Tetrahydroxystilbene glucoside ameliorates diabetic nephropathy in rats: involvement of SIRT1 and TGF-beta1 pathway. Eur J Pharmacol.

[18] Lv L, Shao X, Wang L, Huang D, Ho CT, Sang S (2010). Stilbene glucoside from Polygonum multiflorum Thunb.: a novel natural inhibitor of advanced glycation end product formation by trapping of methylglyoxal. J Agric Food Chem.

[19] Koncsos G, Varga ZV, Baranyai T, Boengler K, Rohrbach S, Li L, Schluter KD, Schreckenberg R, Radovits T, Olah A (2016). Diastolic dysfunction in prediabetic male rats: role of mitochondrial oxidative stress. Am J Physiol Heart Circ Physiol.

[20] Valenti C, Giuliani S, Cialdai C, Tramontana M, Maggi CA (2012). Fasitibant chloride, a kinin B(2) receptor antagonist, and dexamethasone interact to inhibit carrageenan-induced inflammatory arthritis in rats. Br J Pharmacol.

[21] Kho MC, Lee YJ, Park JH, Kim HY, Yoon JJ, Ahn YM, Tan R, Park MC, Cha JD, Choi KM, et al. Fermented Red Ginseng Potentiates Improvement of Metabolic Dysfunction in Metabolic Syndrome Rat Models. Nutrients. 2016;8(6):369. 10.3390/nu8060369 PMC4924210 27322312

[22] Johar DR, Bernstein LH (2017). Biomarkers of stress-mediated metabolic deregulation in diabetes mellitus. Diabetes Res Clin Pract.

[23] Baiceanu A, Mesdom P, Lagouge M, Foufelle F (2016). Endoplasmic reticulum proteostasis in hepatic steatosis. Nat Rev Endocrinol.

[24] Galluzzi L, Vitale I, Abrams JM, Alnemri ES, Baehrecke EH, Blagosklonny MV, Dawson TM, Dawson VL, El-Deiry WS, Fulda S (2012). Molecular definitions of cell death subroutines: recommendations of the nomenclature committee on cell death 2012. Cell Death Differ.

[25] Mi Y, Xiao C, Du Q, Wu W, Qi G, Liu X (2016). Momordin Ic couples apoptosis with autophagy in human hepatoblastoma cancer cells by reactive oxygen species (ROS)-mediated PI3K/Akt and MAPK signaling pathways. Free Radic Biol Med.

[26] Engin A (2017). Non-alcoholic fatty liver disease. Adv Exp Med Biol.

[27] Hu RF, Sun XB (2017). Design of new traditional Chinese medicine herbal formulae for treatment of type 2 diabetes mellitus based on network pharmacology. Chin J Nat Med.

[28] Zheng T, Yang X, Wu D, Xing S, Bian F, Li W, Chi J, Bai X, Wu G, Chen X (2015). Salidroside ameliorates insulin resistance through activation of a mitochondria-associated AMPK/PI3K/Akt/GSK3beta pathway. Br J Pharmacol.

[29] Dong Q, Xing W, Su F, Liang X, Tian F, Gao F, Wang S, Zhang H (2017). Tetrahydroxystilbene glycoside improves microvascular endothelial dysfunction and ameliorates obesity-associated hypertension in obese ZDF rats via inhibition of endothelial autophagy. Cell Physiol Biochem.

[30] Tang W, Li S, Liu Y, Wu JC, Pan MH, Huang MT, Ho CT. Anti-diabetic activities of cis- and trans-2,3,5,4′-tetrahydroxystilbene 2-O-beta-glucopyranoside from *Polygonum multiflorum*. Mol Nutr Food Res. 2017;61(8). 10.1002/mnfr.201600871. 10.1002/mnfr.201600871 28054445

[31] Sun J, Yuan Z, Wang C, Xu H, Zhang L (2005). Pharmacokinetics of stilbene glycoside from Polygonum multiflorum in rats in vivo. Zhong Cao Yao.

